# Anxiety and Coping Strategies in Caregivers of Children With Epidermal Differentiation Disorders: A Case‐Control Study

**DOI:** 10.1155/da/1126242

**Published:** 2026-10-27

**Authors:** Magdalena Fryze, Aldona Pietrzak, Radoslaw Mlak, Bartłomiej Wawrzycki, Katarzyna Wertheim-Tysarowska, Fauve C. A. P. van Veen, Antoni H. Gostynski

**Affiliations:** ^1^ Department of Palliative Medicine Collegium Medicum University of Zielona Góra Zielona Góra 65-046 Poland uz.zgora.pl; ^2^ Department of Dermatology Venereology and Paediatric Dermatology Medical University of Lublin Lublin 20-080 Poland umlub.pl; ^3^ Department of Laboratory Diagnostics Medical University of Lublin Lublin 20-059 Poland umlub.pl; ^4^ Department of Medical Genetics Institute of Mother and Child Warsaw 01-211 Poland imid.med.pl; ^5^ Department of Dermatology Maastricht UMC+ Center of Expertise for Genodermatoses University of Maastricht Maastricht University Medical Center+ Maastricht Netherlands maastrichtuniversity.nl; ^6^ GROW School for Oncology and Reproduction Maastricht University Maastricht Netherlands maastrichtuniversity.nl

**Keywords:** anxiety, caregiver burden, coping strategies, epidermal differentiation disorders (EDDs), psychosocial support

## Abstract

**Background:**

Epidermal differentiation disorders (EDDs) are rare, chronic skin diseases with significant psychosocial impact on patients and families. Their daily management requires complex care, placing caregivers at risk of stress and anxiety. Few studies have systematically examined anxiety and coping strategies in this group.

**Objectives:**

To compare anxiety levels and coping styles between caregivers of children with syndromic or nonsyndromic EDD and caregivers of healthy children.

**Methods:**

In this cross‐sectional case‐control study, 39 caregivers of children with EDD and 39 matched controls were recruited from a dermatology clinic. Anxiety was assessed with the State‐Trait Anxiety Inventory (STAI) and coping styles with the Coping Inventory for Stressful Situations (CISS). Sociodemographic and clinical data were collected.

**Results:**

Caregivers of children with EDDs reported significantly higher trait anxiety than controls (79.5% vs. 53.8%; *p* = 0.017). Task‐oriented coping (TOC) was less frequent among EDD caregivers, with more than half showing low TOC compared with less than one‐third of controls (56.4% vs. 28.6%; *p* = 0.019). High avoidance‐oriented coping (AOC) was observed more often in controls (21.4% vs. 5.6%; *p* = 0.044). Anxiety was greater among caregivers of boys than girls (95.7% vs. 56.2%; *p* = 0.003). Avoidant coping was associated with greater child symptom severity, including pruritus and redness.

**Conclusions:**

Caregivers of children with EDD experience high anxiety and rely less on adaptive coping strategies. These findings emphasize the need for early, family‐centered psychosocial interventions to support caregiver well‐being and improve outcomes for affected children.

## 1. Introduction

In recent years, dermatological experts, psychologists, and dermapsychologists have increasingly directed their attention toward families and caregivers of patients with epidermal differentiation disorders (EDDs) as they are facing a significantly greater burden of parental, social, and educational responsibilities compared to those caring for healthy children without dermatological conditions [1–3]. It is important to emphasize also the considerable expenses incurred by parents in achieving and maintaining a proper skin condition for their children [4]. Emollients, creams, ointments, and hygiene products are a daily necessity, as are additional clothing expenses, as some items of clothing become unwashable after the application of greasy ointments. Additional costs arise for laundry detergents as well as for travel to specialized medical centers that provide treatment services [5]. In some cases, it is necessary to purchase specialized orthopedic strollers to transport nonambulatory children or orthopedic shoes to enable them to move around independently. Expenses may also rise in relation to the psychological support for the affected children and their families [6]. Some patients experience nonspecific yet challenging otolaryngological issues, such as earwax impaction requiring removal or hearing loss [7]. In EDD, certain issues, such as sweat gland dysfunction may prevent patients from participating in physical activity or to cope with higher temperatures [8]. This is due to the general public’s limited awareness of their challenges, the tendency to downplay their disorder, or the lack of appropriate medical assistance stemming from an insufficient understanding of the issue [4].

In recent years, prolonged treatment in medical facilities has increasingly been replaced with a family‐centered care model, in which parents assume a significant share of responsibility for implementing therapeutic recommendations, providing daily care, and monitoring the child’s health status. This approach fosters bonding and allows for better tailoring of treatment to the individual needs of the child. However, it also entails a considerable physical, emotional, and organizational burden on caregivers [9–13].

Psychological and caregiving challenges arise in the case of chronic skin diseases with visible external symptoms, such as nonsyndromic (nEDD), palmoplantar (pEDD), and syndromic EDDs (sEDDs). All these conditions are new classifications of genodermatoses that affect the epidermal layer. These disorders were previously known as ichthyosis, palmoplantar keratoderma, Darier’s disease, or Hailey–Hailey disease, and are characterized by dry and scaly skin, excessive keratinization, pain, and persistent sensory discomfort [14–16]. Treatment is symptomatic and requires daily intensive skincare, which places a significant burden on families. Symptoms may vary depending on the clinical form of the disorder [17–20]. For example, STS‐sEDD (previously known as X‐linked ichthyosis) is associated with large, dark brown scales and may cause mild corneal opacity syndrome; ABHD5‐sEDD (previously known as Chanarin–Dorfman syndrome) is often accompanied by hearing loss; and MBTPS2‐sEDD is characterized, among other symptoms, by pronounced photophobia [14–16]. The complexity and chronicity of these conditions affect not only the patient’s quality of life (QoL) but also intensify the emotional strain experienced by caregivers [17–20].

The visibility of skin lesions makes children affected by EDD particularly vulnerable to social stigmatization, exclusion, and difficulties in peer relationships [1–3]. As a result, parents become not only medical caregivers but also protectors of their child in interactions with the outside world—explaining symptoms, protecting against prejudice, and attempting to mitigate the effects of social stigmatization. Such functioning, although necessary, may lead to chronic overload, increased stress, and a progressive decline in caregivers’ QoL [9, 10, 21–26].

Caring for a child with a chronic, often rare skin disease poses a significant emotional and psychological burden for parents. Long‐term exposure to stressors related to the treatment process, uncertainty of prognosis, and daily caregiving challenges can contribute to the development of anxiety disorders within this group. In the psychological literature, anxiety is understood in two ways: as a state—a temporary emotional reaction to real or perceived threats—and as a trait, meaning a relatively stable tendency to experience emotional tension and worry across various life situations. The coping strategies individuals employ are an important determinant of how they function under stress. According to the transactional theory of stress, coping is defined as a cognitive and behavioral effort aimed at managing specific internal or external demands that are appraised as exceeding the individual’s personal resources [27]. Depending on the nature of the stressor and personality dispositions, individuals may adopt different coping approaches. The literature typically distinguishes three main styles: task‐oriented coping (TOC) (focused on solving the problem), emotion‐oriented coping (involving the experience and expression of emotions), and avoidance‐oriented coping (AOC) (which includes distraction [DIS] or seeking social support) [28–30].

The aim of the present study was to compare the levels of anxiety—as both a state and a trait—in parents of children with sEDD and nEDD and parents of healthy children. In addition, preferred stress coping styles were analyzed, with particular emphasis on avoidance‐oriented strategies. The findings may contribute to a better understanding of the psychological needs of this group of caregivers and support the development of targeted psychosocial interventions for families affected by rare skin diseases.

## 2. Methods

### 2.1. Setting

In this cross‐sectional case‐control study, the study group consisted of caregivers of patients diagnosed with different sEDD/nEDD, who received treatment between 2020 and 2025 at the Dermatology Outpatient Clinic and the Department of Dermatology of the Independent Public Clinical Hospital No. 1 in Lublin. None of the caregivers participating in the study had a diagnosis of sEDD or nEDD. The control group included caregivers of healthy individuals without inherited or chronic skin diseases. They were recruited from patients presenting to the Dermatology Outpatient Clinic for the evaluation of isolated skin lesions. Specifically, the control group consisted of caregivers of children without signs of inflammation, noninflamed verrucae, ephelides, pityriasis versicolor, postinflammatory hyperpigmentation macules, or minor noninflammatory mycotic lesions of the feet. No formal sample size calculation was performed. The sample size was determined by the number of eligible participants who met the inclusion criteria and agreed to participate during the study period.

All participants gave informed consent to participate in the study. The research project was approved by the Bioethics Committee of the Medical University of Lublin (numbers KE 0254/181/2020 and KB 0024/1/01/2025). All procedures were conducted in accordance with applicable guidelines and regulations and based on the principles of the Declaration of Helsinki. Genetic analyses were performed as previously described [31].

The study was conducted using psychometric questionnaires and tools designed to assess the emotional state and personality traits of the participants. The State‐Trait Anxiety Inventory (STAI) was used to analyze the level of anxiety as a state and as a personality trait. The Coping Inventory for Stressful Situations (CISS) was used to assess preferred coping strategies in challenging situations. Furthermore, the participants completed an original questionnaire developed by the authors of the study, which included sociodemographic data and, in the case of caregivers of children with genodermatoses, a description of the specific symptoms presented by patients under their care. The use of these tools enabled a comprehensive psychosocial assessment of the study participants. All questionnaires were properly adapted, and the research procedure was conducted in accordance with established methodological standards.

To minimize potential bias, participants in both groups completed the same validated questionnaires under identical conditions, and predefined inclusion and exclusion criteria were applied.

### 2.2. Description of Research Tools

#### 2.2.1. The STAI

The STAI is a tool used to measure two aspects of anxiety: as a temporary emotional state and as a relatively stable personality trait. It comprises 40 statements divided into two subscales: X1 and X2, with 20 items each. In the X1 subscale, respondents determine the extent to which a given statement reflects their current emotional state, whereas in the X2 subscale, they determine the frequency of a given feeling. The results of both subscales demonstrate reliability levels ranging from 0.70 to 0.90, confirming the high validity of the instrument in measuring anxiety‐related experiences [32].

#### 2.2.2. The CISS

The CISS is a tool used to assess coping strategies in challenging and stressful situations. It includes 48 statements, with responses given on a five‐point Likert scale. There are three main coping strategies: task‐oriented, emotion‐oriented, and avoidance‐oriented. The last one includes two forms: DIS and social diversion. Cronbach’s alpha coefficients for the individual subscales range from 0.78 to 0.90, indicating high internal consistency of the questionnaire [33].

The categorization of results into low, moderate, and high groups for both the STAI and CISS instruments followed the methodological guidelines and original scoring criteria established by the respective scale authors. The analysis therefore conformed to the classification rules outlined in standard interpretation frameworks. Additionally, to improve the clarity of presentation and facilitate interpretation, additional analytical groupings were introduced. Group A compared individuals with low scores to those with combined moderate and high scores, while Group B contrasted low and moderate scores with high scores. This auxiliary approach did not modify the original scale structure but aimed to enhance the transparency and interpretability of the analysis.

#### 2.2.3. Original Questionnaire Developed by the Authors of the Study

An original questionnaire was developed by the authors specifically for this study. It consists of two parts. The first one includes questions on sociodemographic data, such as gender, age, and education. The second part is intended for caregivers of individuals with genodermatoses as it focuses on disease symptoms, including assessment of pain, scale color, skin itching, scaling, redness, infiltration, and the extent of skin lesions. The questionnaire was developed due to the lack of standardized tools allowing for the quantitative assessment of these symptoms.

### 2.3. Statistical Analysis

Statistical analysis of the data collected in the spreadsheet was performed using Statistica software (version 13 PL, Statsoft). The normality of the distribution of continuous variables was assessed using the Shapiro–Wilk test. Due to the non‐normal distribution of continuous variables, relationships between variables were assessed using nonparametric tests in subsequent stages of the analysis. For the same reason, the median was used as a measure of data clustering, while data dispersion was presented using the interquartile range (IQR) and minimum–maximum range. Comparisons of the distribution of continuous variables between the assessed groups were performed using the Mann–Whitney *U* test (when comparing two groups) or the Kruskal–Wallis ANOVA (when comparing more than two groups). Categorical variables were presented using absolute numbers and percentages. Comparisons of categorical variables between the analyzed groups were performed using the Fisher exact test (two compared categories of a variable/group) or the chi‐square test with Yates’ correction (more than two compared categories of a variable/group). For all tests used in the statistical analysis, results with an alpha error (*p*) of less than 0.05 were considered statistically significant.

## 3. Results

### 3.1. Characteristics and Comparison of Demographic, Educational, and Psychological Variables in the Study and Control Groups

A total of 78 participants were included in the study, comprising 39 caregivers of children with EDDs (study group) and 39 caregivers of healthy children (control group). The study and control groups were similar in terms of gender and age, with women comprising the majority in both groups (66.7%). The median [IQR] age were 38 [32–44.75] and 37 [32.25–44.75] years, respectively. There were also no significant differences between the groups regarding the number of children in the household. Caregivers of children with sEDD/nEDD most frequently had one child affected by the disease under their care (61.5%), while 28.2% and 10.3% of caregivers care for two or three children with genodermatosis, respectively. In terms of gender distribution among children with genodermatoses, boys predominated, accounting for 56.4%. A comparison of demographic parameters is presented in Table 1. The genetic findings and characteristics of genetic alterations associated with the diagnosed genodermatosis are presented in Table S1.

**Table 1:** Comparison of selected demographic parameters between study and control groups.

Variable	Study group [n = 39]	Control [n = 39]	*p*
	*n* (%) or median [IQR] (min.–max.)	*n* (%) or median [IQR] (min.–max.)	
Gender of caregiver			
Female	26 (66.7%)	26 (66.7%)	1.0000
Male	13 (33.3%)	13 (33.3%)	—
Age of caregiver (years)	38 [32–44.75] (25–75)	37 [32.25–44.75] (25–75)	0.9243
Gender of child			
Female	17 (43.6%)	22 (56.4%)	0.3650
Male	22 (56.4%)	17 (43.6%)	—
Age of child (years)	16.5 [6–24] (3–54)	8 [5–16.75] (3–45)	0.1173
Total number of children in the household			
1	12 (30.8%)	11 (28.2%)	0.1201
2	12 (30.8%)	21 (53.8%)	—
3	14 (35.9%)	7 (17.9%)	—
5	1 (2.6%)	0 (0%)	—

Abbreviations: IQR, interquartile range; *n*, number.

A significantly higher percentage of individuals with moderate or high levels of trait anxiety (according to the STAI) was recorded in the study group compared to the control group (79.5% vs. 53.8%; *p* = 0.0170). Also, significantly higher proportion of individuals with a low level of TOC, as measured by the CISS, and significantly lower proportion of individuals with a high level of TOC was observed in the study group compared to the control group (56.4% vs. 28.6%; *p* = 0.0192; *p* = 0.0249 and 10.3% vs. 35.7%; *p* = 0.0121, respectively). In turn, a significantly higher proportion of individuals with a high level of AOC was observed in the control group compared to the study group (21.4% vs. 5.6%; *p* = 0.0440). Higher proportion of individuals in the control group presented high level of DIS as compared to the study group but this difference did not reach statistical significance (25% vs. 7.7%; *p* = 0.0516; a result indicating a trend toward statistical significance). A significantly lower percentage of individuals with a high level of social diversion (SOD; CISS) was observed in the study group compared to the control group (10.3% vs. 28.5%; *p* = 0.0145). A comparison of psychological variables in the study and control groups is presented in Table 2.

**Table 2:** Comparison of STAI and CISS results between study and control groups.

Variable	Study group [*n* = 39] *n* (%)	Control [*n* = 39] *n* (%)	*p*
Anxiety (trait) (STAI) (groups)			
Low	8 (20.5%)	18 (46.2%)	0.0170^∗^
Moderate, high	31 (79.5%)	21 (53.8%)	
TOC (CISS)			
High	4 (10.3%)	10 (35.7%)	0.0192^∗^
Moderate	13 (33.3%)	10 (35.7%)	
Low	22 (56.4%)	8 (28.6%)	
TOC (CISS) (groups) A			
Low	22 (56.4%)	8 (28.6%)	0.0249^∗^
Moderate, high	17 (43.6%)	20 (71.4%)	
TOC (CISS) (groups) B			
Low, moderate	35 (89.7%)	18 (64.3%)	0.0121^∗^
High	4 (10.3%)	10 (35.7%)	
EOC (CISS)			
High	6 (15.4%)	2 (7.1%)	0.4598
Moderate	16 (41%)	15 (53.6%)	
Low	17 (43.6%)	11 (39.3%)	
EOC (CISS) (groups) A			
Low	17 (43.6%)	11 (39.3%)	0.7266
Moderate, high	22 (56.4%)	17 (60.7%)	
EOC (CISS) (groups) B			
Low, moderate	33 (84.6%)	26 (92.9%)	0.3085
High	6 (15.4%)	2 (7.1%)	
AOC (CISS)			
High	2 (5.1%)	6 (21.4%)	
Moderate	19 (48.7%)	12 (42.9%)	0.1241
Low	18 (46.2%)	10 (35.7%)	
AOC (CISS) (groups) A			
Low	18 (46.2%)	10 (35.7%)	0.3964
Moderate, high	21 (53.8%)	18 (64.3%)	
AOC (CISS) (groups) B			
Low, moderate	37 (94.9%)	22 (78.6%)	0.0440^∗^
High	2 (5.1%)	6 (21.4%)	
DIS (CISS)			
High	3 (7.7%)	7 (25%)	0.1139
Moderate	19 (48.7%)	9 (32.1%)	
Low	17 (43.6%)	12 (42.9%)	
DIS (CISS) (groups) A			
Low	17 (43.6%)	12 (42.9%)	0.9528
Moderate, high	22 (56.4%)	16 (57.1%)	
DIS (CISS) (groups) B			
Low, moderate	36 (92.3%)	21 (75%)	0.0516
High	3 (7.7%)	7 (25%)	
SOD (CISS) (level)			
High	4 (10.3%)	15 (38.5%)	0.0145^∗^
Moderate	18 (46.2%)	13 (3.3%)	
Low	17 (43.6%)	11 (28.2%)	
SOD (CISS) (groups. v1)			
Low	17 (43.6%)	11 (28.2%)	0.1594
Moderate, high	22 (56.4%)	28 (71.8%)	
SOD (CISS) (groups. v2)			
High	4 (10.3%)	15 (38.5%)	0.0039^∗^
Low, moderate	35 (89.7%)	24 (61.5%)	

Abbreviations: AOC, avoidance‐oriented coping; CISS, Coping Inventory for Stressful Situations; DIS, distraction (subscale of avoidance‐oriented coping); EOC, emotion‐oriented coping; SOD, social diversion (subscale of avoidance‐oriented coping); STAI, State–Trait Anxiety Inventory; TOC, task‐oriented coping.

^∗^ *p* < 0.05.

### 3.2. Detailed Description of Demographic and Clinical Variables in the Study Group

In the study group, autosomal recessive congenital ichthyosis (ARCI) was the most prevalent main diagnosis, accounting for 41% of cases, and all patients presented with the generalized form of the disease. In 89.7% of patients, scales were present on exposed parts of the body. Whole‐body scaling was most common among patients (23.1), and the entire body was covered with scales in 61.5% of patients. The predominant color of the lesions was beige, observed in 41.6% of patients. Ectropion was observed in 28.2%, while hearing loss was observed in 38.9% of patients. A detailed description of clinical variables in the study group is presented in Table 3.

**Table 3:** Detailed description of clinical variables in the study group.

Variable	*N* (%) or Median [IQR] (min.–max.)
Main diagnosis	
ARCI	16 (41%)
Chanarin–Dorfman syndrome	2 (5.1%)
Epidermolytic	3 (7.7%)
EV‐like	2 (5.1%)
Ichthyosiform erythroderma	1 (2.6%)
* MBTPS2*‐sEDD‐IFAP	4 (10.3%)
Netherton syndrome	2 (5.1%)
Sjögren–Larsson syndrome	2 (5.1%)
X‐linked ichthyosis	7 (17.9%)
Genodermatosis type	
ARCI	12 (30.8%)
ARCI‐SICB	1 (2.6%)
ARCI‐LI/PI	2 (5.1%)
ARCI PNPLA1	1 (2.6%)
Epidermolytic	3 (7.7%)
Chanarin–Dorfman syndrome	2 (5.1%)
Ichthyosiform erythroderma	1 (2.6%)
IFAP	4 (10.3%)
EV‐like	2 (5.1%)
X‐linked ichthyosis	7 (17.9%)
Netherton	2 (5.1%)
Sjögren–Larsson syndrome	2 (5.1%)
Extent of the disease	
General	39 (100%)
Scales on exposed parts of the body	
No	4 (10.3%)
Yes	35 (89.7%)
Skin itching	
Median [IQR] (min.–max.)	6 [4–7] (1–10)
Scaling	
Median [IQR] (min.–max.)	6 [5–8] (0–10)
Redness	
Median [IQR] (min.–max.)	4 [2–6] (0–8)
Scaling site	
Dorsum of feet, ankles, calves, knees	2 (5.1%)
Face, body, knee pits	2 (5.1%)
Face, feet, knees, partly abdomen	1 (2.6%)
Face, scalp, lower legs, arms	1 (2.6%)
Forehead, ankles, digits	1 (2.6%)
Forehead, forearms	1 (2.6%)
Front of legs, abdomen	1 (2.6%)
Hands, knees, abdomen, cheeks	2 (5.1%)
Legs	1 (2.6%)
Nape, neck, armpits, groins, palms	1 (2.6%)
Scalp	4 (10.3%)
Scalp, calves	1 (2.6%)
Scalp, elbows, knees, ankles, body	2 (5.1%)
Scalp, legs, abdomen	1 (2.6%)
Torso	2 (5.1%)
Whole body	9 (23.1%)
Whole body except for face	4 (10.3%)
Whole body except for palms and feet	2 (5.1%)
Whole legs, hands	1 (2.6%)
The whole body is covered with scales	
No	15 (38.5%)
Yes	24 (61.5%)
Infiltration	
None	24 (61.5%)
Present	15 (38.5%)
Color of scales	
Beige	5 (12.8%)
Beige and white	2 (5.1%)
Beige, transparent	11 (28.2%)
Brown	7 (17.9%)
Transparent	1 (2.6%)
White	9 (23.1%)
White, brown	1 (2.6%)
Yellow	2 (5.1%)
Yellow, white	1 (2.6%)
Pain (VAS)	1 [0–4] (0–9)
Level of sweating	
No	20 (51.3%)
Yes	19 (48.7%)
Ectropion	
No	28 (71.8%)
Yes	11 (28.2%)
Sleep disorders	
0	12 (30.8%)
1	26 (66.7%)
2	1 (2.6%)
Inability to fully close the eyelids	
No	10 (25.6%)
Yes	29 (74.4%)
The feeling of ear plugging	
No	15 (38.5%)
Yes	24 (61.5%)
Hearing loss	
No	27 (69.2%)
Yes	12 (30.8%)
Performance status	80 [65–93.75] (5–100)
% of body surface covered with lesions	90 [62–98] (10–100)

*Note*: ARCI‐SICB, self‐improving collodion baby, a subtype of autosomal recessive congenital ichthyosis

Abbreviations: ARCI, autosomal recessive congenital ichthyosis; ARCI‐LI/PI, autosomal recessive congenital ichthyosis—lamellar ichthyosis/partial improvement; ARCI‐PNPLA1, autosomal recessive congenital ichthyosis associated with PNPLA1 mutations; EV‐like, epidermodysplasia verruciformis‐like; IFAP, ichthyosis follicularis, alopecia, and photophobia syndrome; IQR, interquartile range; VAS, visual analog scale.

### 3.3. Comparison of Trait Anxiety Levels Among Caregivers Assessed Using the STAI With Regard to Selected Demographic, Educational, and Clinical Variables in the Study Group

A significantly higher proportion of caregivers of male children exhibited moderate to high levels of trait anxiety (according to the STAI) compared to caregivers of female children (95.7% vs. 56.2%; *p* = 0.0031). A comparison of trait anxiety levels among caregivers assessed using the STAI relative to selected demographic, educational, and clinical variables in the study group is presented in Table S2.

### 3.4. Comparison of TOC Levels (Assessed Using the CISS, Group B) Among Caregivers Relative to Selected Demographic, Educational, and Clinical Variables in the Study Group

Children of caregivers with low or moderate levels of TOC (CISS; Group B), had a significantly higher median [IQR] skin itching severity score compared to children of caregivers with a high level of TOC (6 [4–7] vs. 2 [1–4.5]; *p* = 0.0300; Figure 1A). A comparison of TOC levels assessed using the CISS (Group B) among caregivers relative to selected demographic, educational, and clinical variables in the study group is presented in Table S3.

**Figure 1:**
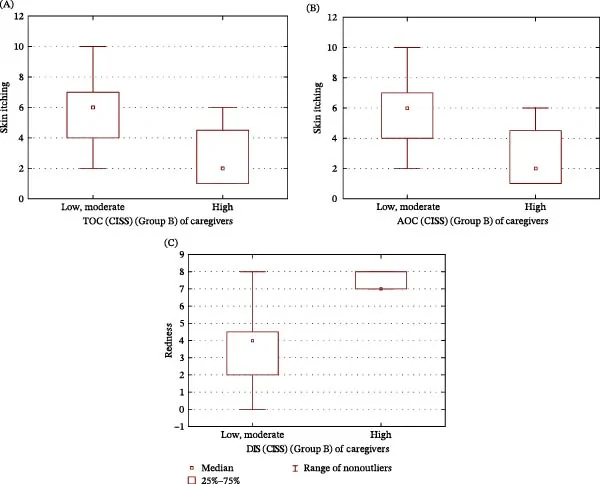
(A) Comparison of the child’s skin itching severity between caregivers with low/moderate and high TOC levels (CISS; Group B). (B) Comparison of the child’s skin itching severity between caregivers with low/moderate and high AOC levels (CISS; Group B). (C) Comparison of the child’s skin redness severity between caregivers with low/moderate and high DIS levels in the study group. AOC, avoidance‐oriented coping; CISS, Coping Inventory for Stressful Situations; DIS, distraction; TOC, task‐oriented coping.

### 3.5. Comparison of AOC Levels Assessed Using the CISS Among Caregivers of Children With Genodermatosis Relative to Selected Demographic, Educational, and Clinical Variables in the Study Group

Children of caregivers with low or moderate levels of AOC (CISS), had a significantly higher median [IQR] skin itching severity score compared to children of caregivers with a high level of AOC (6 [4–7] vs. [1–4.5] 2; *p* = 0.0300; Figure 1B). A comparison of AOC levels assessed using the CISS among caregivers relative to selected demographic, educational, and clinical variables in the study group is presented in Table S4.

### 3.6. Comparison of DIS Levels Assessed Using the CISS (Group B) Among Caregivers Relative to Selected Demographic, Educational, and Clinical Variables in the Study Group

Children of caregivers with high levels of DIS (CISS; Group B) had a significantly higher median redness score compared to children of caregivers with a low or moderate level of DIS (7 [7–7.75] vs. 4 [2–4.5]; *p* = 0.0209; Figure 1C). A comparison of DIS levels assessed using the CISS (Group B) among caregivers relative to selected demographic, educational, and clinical variables in the study group is presented in Table S5.

### 3.7. Comparison of SOD Levels Among Caregivers Assessed Using the CISS Relative to Selected Demographic, Educational, and Clinical Variables in the Study Group

The level of SOD among caregivers did not differ significantly with respect to any of the demographic or clinical variables analyzed. A comparison of the SOD level among caregivers assessed using the CISS relative to selected demographic and clinical variables in the study group is presented in Table S6.

## 4. Discussion

The chronic nature of genodermatoses means that their impact extends beyond the child’s well‐being to include the family and caregivers. Given the long‐term burden associated with caregiving, the coping mechanisms used by parents take on particular significance as they can influence their mental well‐being and the functioning of the entire family system. One of the most important elements of this process is the use of stress coping strategies [1–5, 34–37]. The literature emphasizes that these strategies can serve both adaptive and maladaptive functions, although their effectiveness depends largely on the context and the specific nature of the stressful situation. Actions focused on active problem‐solving, planning, seeking social support, or the constructive use of available resources are generally considered more adaptive. In contrast, strategies based on withdrawal, avoidance, or passive reaction may provide short‐term emotional relief, but in the long term, they are often associated with difficulties in adapting to chronic stress and poorer mental functioning among caregivers. It should be emphasized; however, that the assessment of the effectiveness of individual strategies is not straightforward. Differences in how they are defined and measured, as well as the influence of factors such as the type of stressor, family resources, or the stage of the child’s illness, make it difficult to identify a universally most beneficial way of responding to stress. Despite these limitations, most available studies suggest that strategies focused on addressing the problem are more often associated with better mental well‐being among caregivers, while a predominance of avoidance strategies may hinder the process of adapting to the long‐term challenges of caregiving [34, 38–49].

The results of our study suggest that caregivers of children with genodermatoses are more likely to rely on avoidance‐based coping strategies. Compared to strategies focused on active problem‐solving, such as planning actions, organizing daily responsibilities, or seeking social support, parents more frequently reported a tendency to withdraw, feelings of helplessness, and passivity in the face of stressful situations. These observations are consistent with previous reports regarding parents of children with asthma, cystic fibrosis, congenital heart defects, diabetes, attention‐deficit/hyperactivity disorder (ADHD), or depression, who have also been described as having a tendency to avoid direct confrontation with the difficulties associated with their child’s illness [50–52]. In their study, Alós et al. [45] demonstrated that parents of children with disabilities are more likely than those of healthy children to employ avoidance‐based strategies, including cognitive avoidance, refraining from active engagement, and emotional tension release. These authors suggested that the chronic burden of caring for a child may gradually lead to withdrawal from active problem‐solving and more frequent use of avoidance strategies. Although some of the available studies were based on relatively small samples and qualitative methods, their results remain surprisingly consistent and indicate that parents burdened by long‐term caregiving are more likely to respond with withdrawal, avoidance, or emotional distancing from sources of stress.

Another interesting finding was the association between a greater use of avoidance strategies and poorer child well‐being, including more frequent itching and scratching. Although the nature of this relationship remains unclear, the results may suggest that caregivers’ coping strategies are associated not only with their own mental well‐being but also with the child’s daily experience of the illness. It should be emphasized; however, that the observed relationships are likely multifactorial and should not be interpreted as a simple cause‐and‐effect relationship. It is possible that more severe symptoms of the illness increase the psychological burden on parents and lead to more frequent use of avoidance strategies; however, it seems equally likely that caregivers’ adjustment difficulties may influence how they organize the child’s care. Due to the cross‐sectional design of the study, the direction of this association cannot be determined.

In this context, reports suggesting that coping strategies may have a significant impact on caregivers’ mental health and QoL are particularly noteworthy. Research by Raina [53] showed that more frequent use of stress management strategies was associated with better mental health among caregivers, whereas Khanna et al. [54] observed that a higher prevalence of maladaptive coping strategies was associated with poorer mental functioning. Similar conclusions were presented by Hamama‐Raz and Hamama [55], who indicated that greater flexibility in coping promotes a better QoL in psychological, functional, and overall dimensions.

Also worthy of special attention are the findings of Carona et al. [56], which suggest that the impact of caregiving complexity on parents’ QoL may be partially related to the coping strategies employed. The authors demonstrated that a greater caregiving burden was associated with more frequent use of strategies described as behavioral disengagement, involving a reduction in efforts to cope with the stressor or the abandonment of actions aimed at achieving set goals. These strategies were subsequently associated with a lower QoL for caregivers. These results may suggest that in the process of adapting to a child’s chronic illness, it is not only the caregiving burden itself that matters but also the way in which parents attempt to cope with it. In light of our observations, it can be assumed that coping strategies constitute one of the key elements of the family’s adaptation process to living with a genodermatosis and may be related to both the caregivers’ well‐being and the child’s daily functioning [56]. Further longitudinal studies are needed to clarify the temporal and causal nature of these relationships.

The more frequent use of avoidance strategies observed in our study among caregivers of children with genodermatoses calls for a broader examination of their emotional functioning. The results suggest that the burden associated with daily caregiving may manifest not only in specific coping strategies but also in increased anxiety symptoms. Our study provided additional insights into the psychosocial functioning of caregivers of children with EDD, indicating the presence of a persistent emotional burden associated with the chronic nature of caregiving. Results obtained using the STAI scale suggest that caregivers of children with EDD more frequently exhibited elevated anxiety levels as a trait compared to those of individuals in the control group.

The coexistence of elevated anxiety levels and more frequent use of avoidance strategies may indicate that parents of children with genodermatoses function under conditions of long‐term psychological strain, requiring constant adaptation to the challenges associated with the disease. These observations are consistent with reports on other chronic dermatological diseases, in which elevated levels of stress, anxiety, and depressive symptoms are among the most frequently described psychosocial consequences, affecting both family functioning and the QoL of its members [57, 58].

It is particularly noteworthy that the differences between the groups primarily concerned anxiety as a trait, that is, a relatively stable tendency to respond with anxiety‐rather than merely a transient emotional state triggered by a specific situation. This finding may be associated with persistent emotional strain and the need to constantly adapt to the demands of caring for a child with genodermatosis. This interpretation appears consistent with concepts emphasizing the close relationship between skin function and mental well‐being. Skin is not only the largest and most visible human organ but also an important element shaping relationships with the environment and self‐perception. In the case of diseases characterized by visible skin lesions, this may contribute to heightened concerns regarding the child’s social functioning, acceptance by peers, and futureQoL.

Furthermore, our findings may also be considered within the broader context of chronic caregiving stress and psychological adaptation to long‐term illness. Previous research has shown that parents of children with disabilities may experience elevated levels of death anxiety and existential concerns related to their child’s health, future functioning, and long‐term prognosis [59]. This higher level of death anxiety was associated with both adaptive and maladaptive coping attitudes, while psychosocial support emerged as an important factor related to parental adjustment. These findings underscore the complex relationship between anxiety, coping mechanisms, and the psychological well‐being among caregivers of children with chronic conditions. Although death anxiety was not assessed in the present study, the elevated anxiety levels and greater reliance on AOC strategies observed among caregivers of children with genodermatoses may reflect broader psychological adaptation processes associated with the long‐term caregiving burden.

Another interesting observation was the relationship between anxiety levels and the child’s gender. Parents of boys were more likely to report moderate or high levels of anxiety than those of parents of girls. However, this finding should be interpreted with caution given the relatively small sample size and the exploratory nature of the analysis. Although the underlying reasons remain unclear, it is possible that cultural and social expectations related to gender roles influence how parents experience the illness. A visible, chronic, and potentially stigmatizing illness may be perceived as a particular challenge to socially‐entrenched notions regarding boys’ independence, physical ability, or resilience. However, the present study was not designed to investigate these mechanisms directly, and therefore, no definitive conclusions can be drawn regarding the reasons underlying this association. Rather, this observation should be considered preliminary and hypothesis‐generating, warranting further investigation in larger cohorts. Future studies may help determine whether the child’s gender influences caregivers’ emotional experiences and identify factors that may contribute to this relationship.

Our findings also have significant clinical and organizational implications. First, specialists treating children with genodermatoses should routinely include an assessment of parents’ mental well‐being as part of comprehensive care. Early identification of elevated anxiety levels, maladaptive coping strategies, or tendencies toward social withdrawal could enable families to be referred more quickly to appropriate forms of psychological support. Second, interventions should go beyond treating dermatological symptoms and address the psychosocial needs of the entire family. In this context, psychoeducational programs aimed at developing adaptive coping strategies appear particularly promising. Findings from studies conducted among caregivers of children with other chronic conditions, including epilepsy, indicate that such interventions can effectively reduce the burden of caregiving, levels of anxiety, and the use of maladaptive coping strategies while simultaneously reinforcing more constructive and flexible ways of responding to stress. This suggests that similar interventions could potentially benefit the families of children with genodermatoses as well [60].

Despite the valuable insights provided by our study, several limitations should be noted. First, while the sample size was sufficient to detect statistically significant differences, it may limit the generalizability of the results to all caregivers of children with genodermatoses. A significant strength of the study; however, is its coverage of a broad spectrum of EDD, which enhances its external validity and supports the applicability of the findings to diverse clinical presentations. Second, the cross‐sectional nature of the study precludes drawing causal conclusions regarding the relationship between coping strategies, caregivers’ anxiety levels, and children’s health outcomes. Consequently, the observed associations may reflect bidirectional relationships or the influence of unmeasured factors, and the temporal sequence of these variables cannot be established. Elucidating these relationships requires longitudinal studies. Third, the use of self‐report methods alone may have led to reporting biases, particularly regarding sensitive issues such as mental health or coping styles. Future studies could benefit from combining self‐report data with clinical assessment or objective behavioral measures. A further limitation of this study is the difference in the ages of children between the study and control groups. Although this difference did not reach statistical significance, caring for children at different developmental stages may involve distinct psychological, emotional, and practical demands. For example, caregiving challenges associated with adolescence may differ substantially from those related to caring for younger children. Consequently, age‐related factors may have influenced caregivers’ experiences and coping strategies and should be considered a potential confounding factor when interpreting the findings. Also, a relatively large number of statistical comparisons were performed without formal adjustment for multiple testing, which increases the risk of type I error. Consequently, some of the observed associations may represent chance findings and should be interpreted with caution until confirmed in larger independent samples. Second, although detailed descriptive statistics were provided to facilitate the interpretation of the results, effect size measures were not systematically reported. Therefore, the magnitude and practical significance of the observed differences should be interpreted cautiously. However, to reduce the risk of selection bias, the study was conducted prospectively by recruiting eligible participants sequentially, which minimized the likelihood of systematic differences between caregivers included and excluded from the study.

## 5. Conclusions

In conclusion, our study demonstrates that caregivers of children with genodermatoses experience significant and sustained anxiety, often relying on maladaptive coping strategies that may negatively influence both their own well‐being and the child’s disease outcomes. Importantly, we observed a preliminary association between caregiver anxiety and child gender. While the underlying mechanisms remain unclear, this finding raises the possibility that sociocultural expectations may influence parental responses to chronic visible illnesses and merit further investigation in larger cohorts. Our findings underscore the urgent need for comprehensive, family‐centered care models that integrate psychological support for caregivers alongside dermatological treatment for children. Addressing the psychosocial burden of caregiving in genodermatoses is not only essential for improving parental QoL but may also enhance treatment adherence and clinical outcomes for affected children. Further research should focus on developing tailored interventions that support caregiver resilience, foster adaptive coping strategies, and address the unique challenges associated with visible, stigmatizing chronic skin conditions.

## Author Contributions

Conceptualization: Aldona Pietrzak, Magdalena Fryze, Radoslaw Mlak, and Antoni H. Gostynski. Data curation and formal analysis: Radoslaw Mlak, Aldona Pietrzak, Magdalena Fryze, Bartłomiej Wawrzycki, and Katarzyna Wertheim‐Tysarowska. Funding acquisition, project administration: Aldona Pietrzak. Investigation: Aldona Pietrzak, Magdalena Fryze, and Bartłomiej Wawrzycki. Methodology: Magdalena Fryze, Katarzyna Wertheim‐Tysarowska, and Radoslaw Mlak. Resources: Aldona Pietrzak and Radoslaw Mlak. Supervision: Aldona Pietrzak and Magdalena Fryze. Validation: Fauve C. A. P. van Veen. Writing – original draft: Magdalena Fryze, Aldona Pietrzak, Radoslaw Mlak, Fauve C. A. P. van Veen, Katarzyna Wertheim‐Tysarowska, Fauve C. A. P. van Veen, and Antoni H. Gostynski. Writing – review and editing: Aldona Pietrzak, Radoslaw Mlak, Magdalena Fryze, Bartłomiej Wawrzycki, Katarzyna Wertheim‐Tysarowska, Fauve C. A. P. van Veen, and Antoni H. Gostynski.

## Funding

This study was funded by the Medical University of Lublin, Poland (Grant DA 168).

## Disclosure

All the authors reviewed the manuscript.

## Conflicts of Interest

The authors declare no conflicts of interest.

## Supporting Information

Additional supporting information can be found online in the Supporting Information section.

## Supporting information


**Supporting Information** The Supporting Information includes six tables providing additional data on the study cohort and the analyses performed. Table S1: Presents the genetic findings in the study cohort. Table S2: Provides additional analyses of trait anxiety assessed using the STAI according to selected demographic, educational, and clinical variables. Tables S3–S6: Present additional analyses of coping strategies assessed using the CISS, including TOC, AOC, DIS, and overall stress coping strategies, according to selected demographic, educational, and clinical variables.

## Data Availability

The datasets generated and/or analyzed during the current study are available from the corresponding author upon reasonable request.
