# Supplementary material for: Anxiety and Coping Strategies in Caregivers of Children With Epidermal Differentiation Disorders: A Case‐Control Study
Source: Depress Anxiety. 2026 Oct 27;2026:1126242. doi: 10.1155/da/1126242 (PMC13711021; doi:10.1155/da/1126242)
Supplement: Supplementary file 1 [file DA-2026-1126242-s001.docx]

**Table S1.** Genetic findings in the study cohort

| **Parent no** | **Sex of the child** | **Category according to 2009 classification of ichthyosis (REF) [60]** | **Name according to 2025 classification of epidermal differentiation disorder(REF) [61-62]** | **Genotype** |
| --- | --- | --- | --- | --- |
| 1 | boy | ARCI | *ALOX12B- nEDD* | NM_001139.2:c.1562A>G(;)808A>C NP_001130.1: p.(Tyr521Cys)(;)(Asn270His) |
| 1 |  |  |  |  |
| 2 | girl | ARCI (ARCI-SICB) | *ALOX12B- nEDD* | NM_001139.2: c.1207C>T(;)1790C>A NP_001130.1: p.(His403Tyr)(;)(Ala597Glu) |
| 3 | girl | Netherton | *SPINK5-sEDD* | NM_006846.4:c.1530C>A(;)(1530C>A) NP_006837.2:p.(Cys510Ter)(;)(Cys510Ter) |
| 3 |  |  |  |  |
| 4 | girl | ARCI | *CYP4F22- nEDD* | NM_173483.3: c.[667C>T];[(667C>T)]   NP_775754.2: p.(Gln223Ter)(;)(Gln223Ter) |
| 5 | girl | ARCI | *ALOX12B- nEDD* | NM_001139.2: c.1562A>G (;)1790C>A ; NP_001130.1: p.(Tyr521Cys)(;)(Ala597Glu) |
| 6 | girl | ARCI | *TGM1- nEDD* | NM_000359:c.[377G>A];[1403-355_2225+1704dup] NP_000350.1: p.[Arg126His];[=] |
| 6 |  |  |  |  |
| 7 | boy | EV-like | *nEDD* | during diagnosis |
| 7 |  |  |  |  |
| 8 | girl | Chanarin-Dorfman syndrome | *ABHD5-sEDD* | NM_001355186.2:c.19G>T(;)254G>A NP_001342115.1:p.(Glu7Ter)(;)Gly85Glu |
| 8 |  |  |  |  |
| 9 | boy | X-linked ichthyosis | *STS-sEDD* | (hg38) NG_021472.2:g.[7050159-7259569dup];[0] |
| 9 |  |  |  |  |
| 10 | girl | Sjögren-Larsson syndrome | *ALDH3A2-sEDD* | NM_000382.2:c.[733G>A];[733G>A] NP._000373.1:p.[Asp245Asn];[Asp245Asn] |
| 10 |  |  |  |  |
| 11 | girl | epidermolytic | *KRT10- nEDD- epidermolytic* | NM_000421.5:[467G>A];[=] NP_000412.4:p.[Arg156His];[=] |
| 11 |  |  |  |  |
| 12 | boy | X-linked ichthyosis | *STS-sEDD* | NC_000023.10:g.[(?_7137717)_(7252168_?)del];[0] |
| 12 |  |  |  |  |
| 13 | boy | ARCI | *ALOXE3- nEDD* | NM_021628.2 : c.680+1G>A(;)842delG NP_067641.2:p.(?);(Gly281ValfsTer38) |
| 13 |  |  |  |  |
| 14 | boy | IFAP | *MBTPS2-sEDD* | NM_015884.4: c.[1001G>A];[0] NP_056968.1 p.[(Cys334Tyr)];[0] |
| 14 |  |  |  |  |
| 15 | boy | ARCI | *ALOX12B- nEDD* | NM_001139.2: c.[1562A>G];[2094C>A];  NP_001130.1: p.[Tyr521Cys];[Ser698Arg] |
| 15 |  |  |  |  |
| 16 | girl | ARCI | *TGM1- nEDD* | NM_000359.2: c.788G>A(;)1135G>C NP_000350.1: p.(Trp263Ter)(;)(Val379Leu) |
| 17 | boy | X-linked ichthyosis | *ALOXE- nEDD 3* | NM_021628.2: c.[700C>T];[(700C>T)] NP_067641.2: p.[Arg234Ter];[(Arg234Ter)] |
| 18 | girl | epidermolytic | *KRT10- nEDD-epidermolytic* | NM_000421.3:c.[467G>A];[=]. NP_000412.3:p.[Arg156His];[=] |
| 19 | boy | X-linked ichthyosis | *STS-sEDD* | NC_000023.10:g.[(?_7137717)_(7252168_?)del];[0] |
| 20 | girl | ARCI | *TGM1 - nEDD* | NM_000359.2: c.[377G>A];[1135G>C] NP_000350.1: p.[Arg126His];[Val379Leu] |
| 21 | girl | ARCI | *ALOX12B- nEDD* | NM_001139.2: c.[2036G>A];[2060A>G]  NP_001130.1: p.[Arg679His];[Tyr687Cys] |
| 22 | boy | ARCI | *PNPLA1- nEDD* | NM_001145717.1:c.[275del];[301A>G] NP_001139189.2:p.[(Pro925ArgfsTer8)];[(Arg101Gly)] |
| 23 | boy | ichthyosiform erythroderma | *Unspecified- nEDD* | declared by mother |
| 24 | boy | IFAP | *Unspecified- sEDD* | NM_015884.4: c.[1001G>A];[0]  NP_056968.1 p.[(Cys334Tyr)];[0] |
| 25 | boy | ARCI | *Unspecified- nEDD* | declared by mother |
| 26 | boy | X-linked ichthyosis | *Unspecified -sEDD* | declared by mother |

**Abbreviations: ARCI - Autosomal Recessive Congenital Ichthyosis; IFAP - Ichthyosis Follicularis, Atrichia and Photophobia (IFAP) syndrome; nEDD – non-syndromic Epidermal Differentiation Disorder; sEDD – syndromic Epidermal Differentiation Disorder**

**Table S2.** Comparison of trait anxiety levels among caregivers assessed using the STAI with regard to selected demographic, educational, and clinical variables in the study group.

| **Variable** | **Trait anxiety (STAI) of caregivers** | | ***p*** |
| --- | --- | --- | --- |
|  | **N (%)**  **or median**  **[IQR]**  **(min.-max.)** | |  |
|  | **Low** | **Moderate, High** |  |
| **Gender**  Female  Male | 6 (23.1%)  2 (15.4%) | 20 (76.9%)  11 (84.6%) | 0.5799 |
| **Age of caregivers [years]** | 36  [31-44]  (25-48) | 39  [32-46.25]  (26-75) | 0.5081 |
| **Total number of children**  1  2  3  4  5 | 4 (33.3%)  1 (8.3%)  2 (14.3%)  0 (0%)  1 (100%) | 8 (66.7%)  11 (91.7%)  12 (85.7%)  0 (0%)  0 (0%) | 0.0893 |
| **Total number of children with genodermatosis**  1  2  3 | 6 (25%)  0 (0%)  2 (50%) | 18 (75%)  11 (100%)  2 (50%) | 0.0718 |
| **Gender of child with genodermatosis**  Male  Female | 1 (4.3%)  7 (43.7%) | 22 (95.7%)  9 (56.2%) | 0.0031* |
| **Age of child with genodermatosis [years]** | 7  [4.5-17]  (4-24) | 13  [5-21.75]  (3-41) | 0.4019 |
| **Main diagnosis**  ARCI  Other | 3 - 20  5 (21.7%) | 12 (80%)  18 (78.3%) | 0.8991 |
| **Scales on exposed parts of the body**  No  Yes | 1 (25%)  7 (20%) | 3 (75%)  28 (80%) | 0.8169 |
| **Skin itching** | 6  [3.50-7.50]  (3-10) | 5  [4- 7]  (1- 10) | 0.7258 |
| **Scaling** | 7  [6-9.50]  (2-10) | 6  [3.50-8]  (0- 10) | 0.1943 |
| **Redness** | 4  [2.50-6.50]  (1-8) | 4  [2- 6]  (0- 8) | 0.5707 |
| **Scaling site**  Whole body  Other | 2 (22.2%)  6 (20%) | 7 (77.8%)  24 (80%) | 0.8863 |
| **The whole body is covered with scales**  No  Yes | 3 - 20  5 (20.8%) | 12 (80%)  19 (79.2%) | 0.9506 |
| **Infiltration**  None  Present | 5 (20.8%)  3 - 20 | 19 (79.2%)  12 (80%) | 0.9506 |
| **Color of scales**  Beige  Other | 4 (22.2%)  4 (19%) | 14 (77.8%)  17 (81%) | 0.8091 |
| **Pain (VAS)** | 0  [0-2.50]  (0-5) | 1  [0- 4]  (0- 9) | 0.3318 |
| **Level of sweating**  No  Yes | 5 (25%)  3 (15.8%) | 15 (75%)  16 (84.2%) | 0.4822 |
| **Ectropion**  No  Yes | 5 (17.9%)  3 (27.3%) | 23 (82.1%)  8 (72.7%) | 0.5177 |
| **Sleep disorders**  0  1, 2 | 2 (16.7%)  6 (22.2%) | 10 (83.3%)  21 (77.8%) | 0.6955 |
| **Inability to fully close the eyelids**  No  Yes | 4 (40%)  4 (13.8%) | 6 (60%)  25 (86.2%) | 0.0806 |
| **The feeling of ear plugging**  No  Yes | 2 (13.3%)  6 (25%) | 13 (86.7%)  18 (75%) | 0.3862 |
| **Hearing loss**  No  Yes | 6 (22.2%)  2 (16.7%) | 21 (77.8%)  10 (83.3%) | 0.6955 |
| **Performance status** | 82.5  [47.50-90]  (30-95) | 80  [65-95]  (5-100) | 0.9160 |
| **% of body surface covered with lesions** | 70  [64-90.50]  (40-95) | 94  [62-100]  (10-100) | 0.1316 |

ARCI, Autosomal Recessive Congenital Ichthyosis; IQR, Interquartile Range; STAI, State-Trait Anxiety Inventory; VAS, Visual Analogue Scale.

**Table S3.** Comparison of TOC levels assessed using the CISS (group B) among caregivers relative to selected demographic, educational, and clinical variables in the study group.

| **Variable** | **TOC (CISS) (group B) of caregivers** | | ***p*** |
| --- | --- | --- | --- |
|  | **N (%)**  **or Median**  **[IQR]**  **(min.-max.)** | |  |
|  | **Low, Moderate** | **High** |  |
| **Gender**  Female  Male | 24 (92.3%)  11 (31.4%) | 2 (7.7%)  2 (15.4%) | 0.4613 |
| **Age of caregivers [years]** | 38  [32-44.75]  (25-75) | 37.5  [33-43.50]  (30-48) | 0.8711 |
| **Age [years]**  <38  ≥38 | 16 (88.9%)  19 (90.5%) | 2 (11.1%)  2 (9.5%) | 0.8723 |
| **Education**  Higher  Other | 14 (87.5%)  21 (91.3%) | 2 (12.5%)  2 (8.7%) | 0.7038 |
| **The total number of children**  1  2  3  4  5 | 10 (83.3%)  11 (84.6%)  11 (100%)  2 (100%)  1 (100%) | 2 (16.7%)  2 (15.4%)  0 (0%)  0 (0%)  0 (0%) | 0.6434 |
| **Gender of child with genodermatosis**  Male  Female | 20 (87%)  15 (93.7%) | 3 (13%)  1 (6.2%) | 0.4972 |
| **Age of child with genodermatosis [years]** | 1  [1-2]  (1-3) | 1.5  [1-2]  (1-2) | 0.7884 |
| **Main diagnosis**  ARCI  Other | 13 (86.7%)  21 (91.3%) | 2 (13.3%)  2 (8.7%) | 0.6532 |
| **Scales on exposed parts of the body**  No  Yes | 4 (100%)  31 (88.6%) | 0 (0%)  4 (11.4%) | 0.4811 |
| **Skin itching** | 6  [4-7]  (2-10) | 2  [1-4.5]  (1-6) | 0.0300* |
| **Scaling** | 6  [5-8]  (0-10) | 5.5  [3-9]  (3-10) | 0.9628 |
| **Redness** | 4  [2-6]  (1-8) | 2  [0-5]  (0-6) | 0.2478 |
| **Scaling site**  Whole body  Other | 8 (88.9%)  27 (90%) | 1 (11.1%)  3 (10%) | 0.9242 |
| **The whole body is covered with scales**  No  Yes | 23 (95.8%)  12 (80%) | 1 (4.2%)  3 (20%) | 0.1176 |
| **Infiltration**  None  Present | 21 (87.5%)  14 (93.9%) | 3 (12.5%)  1 (6.7%) | 0.5642 |
| **Color of scales**  Beige  Other | 16 (88.9%)  19 (90.5%) | 2 (11.1%)  2 (9.5%) | 0.8723 |
| **Pain (VAS)** | 1  [0-4]  (0-9) | 0  [0-4.5]  (0-9) | 0.4797 |
| **Level of sweating**  No  Yes | 18 (90%)  17 (89.5%) | 2 (10%)  2 (10.5%) | 0.9574 |
| **Ectropion**  No  Yes | 25 (89.5%)  10 (90.9%) | 3 (10.7%)  1 (9.1%) | 0.8820 |
| **Sleep disorders**  0  1. 2 | 11 (91.7%)  24 (88.9%) | 1 (8.3%)  3 (11.1%) | 0.7945 |
| **Inability to fully close the eyelids**  No  Yes | 8 (80%)  27 (93.1%) | 2 (20%)  2 (6.9%) | 0.2450 |
| **The feeling of ear plugging**  No  Yes | 15 (100%)  20 (83.3%) | 0 (0%)  4 (16.7%) | 0.0995 |
| **Hearing loss**  No  Yes | 24 (88.9%)  11 (91.7%) | 3 (11.1%)  1 (8.3%) | 0.7945 |
| **Performance status** | 85  [61.25-95]  (5-100) | 72.5  [65-80]  (65-80) | 0.4264 |
| **% of the body surface covered with lesions** | 90  [60-99.5]  (10-100) | 90  [87.5-93]  (86-95) | 0.9813 |

**Table S4.** Comparison of AOC levels assessed using the CISS among caregivers relative to selected demographic, educational, and clinical variables in the study group.

| **Variable** | **AOC (CISS) (gr B) of caregivers** | | ***p*** |
| --- | --- | --- | --- |
|  | **N (%)**  **or Median**  **[IQR]**  **(min.-max.)** | |  |
|  | **Low, Moderate** | **High** |  |
| **Gender**  Female  Male | 24 (92.3%)  13 (100%) | 2 (7.7%)  0 (0%) | 0.3109 |
| **Age of caregivers [years]** | 38  [32-44.75]  (25-75) | 37.5  [33-43.5]  (30-48) | 0.8711 |
| **Age [years]**  <38  ≥38 | 17 (94.4%)  20 (95.2%) | 1 (5.6%)  1 (4.8%) | 0.9120 |
| **Education**  Higher  Other | 14 (87.5%)  23 (100%) | 2 (12.5%)  0 (0%) | 0.0857 |
| **The total number of children**  1  2  3  4  5 | 12 (100%)  13 (100%)  9 (81.8%)  2 (100%)  1 (100%) | 0 (0%)  0 (0%)  2 (18.2%)  0 (0%)  0 (0%) | 0.2518 |
| **Gender of child with genodermatosis**  Male  Female | 21 (91.3%)  16 (100%) | 2 (8.7%)  0 (0%) | 0.2319 |
| **Age of child with genodermatosis [years]** | 6.5  [4-18]  (3-41) | 11  [11-20]  (11-23) | 0.3270 |
| **Main diagnosis**  ARCI  Other | 14 (93.3%)  22 (95.7%) | 1 (6.7%)  1 (4.3%) | 0.7575 |
| **Scales on exposed parts of the body**  No  Yes | 4 (100%)  33 (94.3%) | 0 (0%)  2 (5.7%) | 0.6280 |
| **Skin itching** | 6  [4-7]  (2-10) | 2  [1-4.5]  (1-6) | 0.0300* |
| **Scaling** | 6  [5-8]  (0-10) | 5.5  [3-9]  (3-10) | 0.9627 |
| **Redness** | 4  [2-6]  (1-8) | 2  [0-5]  (0-6) | 0.2478 |
| **Scaling site**  Whole body  Other | 9 (100%)  28 (93.3%) | 0 (0%)  2 (6.7%) | 0.4325 |
| **The whole body is covered with scales**  No  Yes | 22 (91.7%)  15 (100%) | 2 (8.3%)  0 (0%) | 0.2572 |
| **Infiltration**  None  Present | 22 (91.7%)  15 (100%) | 2 (8.3%)  0 (0%) | 0.2572 |
| **Color of scales**  Beige  Other | 17 (94.4%)  20 (95.2%) | 1 (5.6%)  1 (4.8%) | 0.9120 |
| **Pain (VAS)** | 1  [0-4]  (0-9) | 0  [0-4.5]  (0-9) | 0.4797 |
| **Level of sweating**  No  Yes | 19 (95%)  18 (94.7%) | 1 (5%)  1 (5.3%) | 0.9707 |
| **Ectropion**  No  Yes | 27 (96.4%)  10 (90.9%) | 1 (3.6%)  1 (9.1%) | 0.4876 |
| **Sleep disorders**  0  1. 2 | 11 (91.7%)  26 (96.3%) | 1 (8.3%)  1 (3.7%) | 0.5504 |
| **Inability to fully close the eyelids**  No  Yes | 10 (100%)  27 (93.1%) | 0 (0%)  2 (6.9%) | 0.4000 |
| **The feeling of ear plugging**  No  Yes | 14 (93.3%)  23 (95.8%) | 1 (6.7%)  1 (4.2%) | 0.7339 |
| **Hearing loss**  No  Yes | 26 (96.3%)  11 (91.7%) | 1 (3.7%)  1 (8.3%) | 0.5504 |
| **Performance status** | 85  [61.25-95]  (5-100) | 72.5  [65-80]  (65-80) | 0.4264 |
| **% of the body surface covered with lesions** | 90  [60-99.5]  (10-100) | 90  [87.5-93]  (86-93) | 0.9814 |

**Table S5.** Comparison of DIS levels assessed using the CISS (group B) among caregivers relative to selected demographic, educational, and clinical variables in the study group.

| **Variable** | **DIS (CISS) (group B) of caregivers** | | ***p*** |
| --- | --- | --- | --- |
|  | **N (%)**  **or Median**  **[IQR]**  **(min.-max.)** | |  |
|  | **Low, Moderate** | **High** |  |
| **Gender**  Female  Male | 24 (92.3%)  12 (92.3%) | 2 (7.7%)  1 (7.7%) | 1.0000 |
| **Age of caregivers [years]** | 38.5  [32-44.5]  (25-75) | 35  [32.75-65]  (32-75) | 0.8742 |
| **Age [years]**  <38  ≥38 | 16 (88.9%)  20 (95.2%) | 2 (11.1%)  1 (4.8%) | 0.4640 |
| **Education**  Higher  Other | 14 (87.5%)  2 (95.7%) | 2 (12.5%)  1 (4.3%) | 0.3536 |
| **The total number of children**  1  2  3  4  5 | 12 (100%)  13 (100%)  9 (81.8%)  1 (50%)  1 (100%) | 0 (0%)  0 (0%)  2 (18.2%)  1 (50%)  0 (0%) | 0.0633 |
| **Gender of child with genodermatosis**  Male  Female | 20 (87%)  16 (100%) | 3 (13%)  0 (0%) | 0.1378 |
| **Age of child with genodermatosis[years]** | 9.5  [5-18]  (3-41) | 3  [3-31.5]  (3-41) | 0.3554 |
| **Main diagnosis**  ARCI  Other | 13 (86.7%)  22 (95.7%) | 2 (13.3%)  1 (4.3%) | 0.3218 |
| **Scales on exposed parts of the body**  No  Yes | 4 (100%)  32 (91.4%) | 0 (0%)  3 (8.6%) | 0.5475 |
| **Skin itching** | 5  [3.5-7]  (1-10) | 7  [7-7.75]  (7-8) | 0.1051 |
| **Scaling** | 6  [4-8]  (0-10) | 6  [6-9]  (6-10) | 0.4567 |
| **Redness** | 4  [2-4.5]  (0-8) | 7  [7-7.75]  (7-8) | 0.0209* |
| **Scaling site**  Whole body  Other | 28 (93.3%)  8 (88.9%) | 2 (6.7%)  1 (11.1%) | 0.6649 |
| **The whole body is covered with scales**  No  Yes | 22 (91.7%)  14 (93.3%) | 2 (8.3%)  1 (6.7%) | 0.8512 |
| **Infiltration**  None  Present | 21 (87.5%)  15 (100%) | 3 (12.5%)  0 (0%) | 0.1595 |
| **Color of scales**  Beige  Other | 16 (88.9%)  20 (95.2%) | 2 (11.1%)  1 (4.8%) | 0.4640 |
| **Pain (VAS)** | 1  [0-4]  (0-9) | 1  [0.25-1]  (0-1) | 0.6977 |
| **Level of sweating**  No  Yes | 19 (95%)  17 (89.5%) | 1 (5%)  2 (10.5%) | 0.5228 |
| **Ectropion**  No  Yes | 27 (96.4%)  9 (81.8%) | 1 (3.6%)  2 (18.2%) | 0.1283 |
| **Sleep disorders**  0  1. 2 | 11 (91.7%)  25 (92.6%) | 1 (8.3%)  2 (7.4%) | 0.9212 |
| **Inability to fully close the eyelids**  No  Yes | 10 (100%)  26 (89.7%) | 0 (0%)  3 (10.3%) | 0.2960 |
| **The feeling of ear plugging**  No  Yes | 14 (93.3%)  22 (91.7%) | 1 (6.7%)  2 (8.3%) | 0.8512 |
| **Hearing loss**  No  Yes | 26 (96.3%)  10 (83.3%) | 1 (3.7%)  2 (16.7%) | 0.1663 |
| **Performance status** | 82.5  [65-95]  (5-100) | 65  [61.25-65]  (60-65) | 0.1581 |
| **% of the body surface covered with lesions** | 90.5  [60-98]  (10-100) | 90  [90-97.5]  (90-100) | 0.4897 |

**Table S6.** Comparison of the level of stress coping strategies among caregivers assessed using the CISS relative to selected demographic, educational, and clinical variables in the study group.

| **Variable** | **SOD (CISS) of caregivers** | | ***p*** |
| --- | --- | --- | --- |
|  | **N (%)**  **or Median**  **[IQR]**  **(min.-max.)** | |  |
|  | **Low, Moderate** | **High** |  |
| **Gender**  Female  Male | 4 (100%)  31 (88.6%) | 0 (0%)  4 (11.4%) | 0.4811 |
| **Age of caregivers [years]** | 36  [31-44]  (25-48) | 39  [32-46.25]  (26-75) | 0.5081 |
| **Age [years]**  <38  ≥38 | 16 (88.9%)  19 (90.5%) | 2 (11.1%)  2 (9.5%) | 0.8723 |
| **Education**  Higher  Other | 15 (93.7%)  20 (87%) | 1 (6.2%)  3 (13%) | 0.4972 |
| **Total number of children**  1  2  3  4  5 | 5 (41.7%)  4 (33.3%)  8 (57.1%)  0 (0%)  0 (0%) | 7 (58.3%)  8 (66.7%)  6 (42.9%)  0 (0%)  1 (100%) | 0.5030 |
| **Gender of child with genodermatosis**  Male  Female | 20 (87%)  15 (93.7%) | 3 (13%)  1 (6.2%) | 0.4972 |
| **Age of child with genodermatosis [years]** | 7  [4.75-23]  (3-41) | 12  [5-18]  (3-41) | 0.6087 |
| **Main diagnosis**  ARCI  Other | 15 (100%)  19 (82.9%) | 0 (0%)  4 (17.4%) | 0.0920 |
| **Scales on exposed parts of the body**  No  Yes | 4 (100%)  31 (88.6%) | 0 (0%)  4 (11.4%) | 0.4811 |
| **Skin itching** | 6  [3.50-7.50]  (3-10) | 5  [4-7]  (1-10) | 0.7258 |
| **Scaling** | 7  [6-9.50]  (2-10) | 6  [3.50-8]  (0-10) | 0.1943 |
| **Redness** | 4  [2.50-6.50]  (1-8) | 4  [2-6]  (0- 8) | 0.5707 |
| **Scaling site**  Whole body  Other | 9 (100%)  26 (86.7%) | 0 (0%)  4 (13.3%) | 0.2537 |
| **The whole body is covered with scales**  No  Yes | 15 (100%)  20 (83.3%) | 0 (0%)  4 (16.7%) | 0.0995 |
| **Infiltration**  None  Present | 23 (95.8%)  12 (80%) | 1 (4.2%)  3 - 20 | 0.1176 |
| **Color of scales**  Beige  Other | 17 (94.4%)  18 (85.7%) | 1 (5.6%)  3 (14.3%) | 0.3765 |
| **Pain (VAS)** | 0  [0-2.50]  (0-5) | 1  [0-4]  (0-9) | 0.3318 |
| **Level of sweating**  No  Yes | 19 (95%)  16 (84.2%) | 1 (5%)  3 (15.8%) | 0.2732 |
| **Ectropion**  No  Yes | 24 (85.7%)  11 (100%) | 4 (14.3%)  0 (0%) | 0.1915 |
| **Sleep disorders**  0  1, 2 | 11 (91.7%)  24 (88.9%) | 1 (8.3%)  3 (11.1%) | 0.7945 |
| **Inability to fully close the eyelids**  No  Yes | 10 (100%)  25 (86.2%) | 0 (0%)  4 (13.8%) | 0.2211 |
| **The feeling of ear plugging**  No  Yes | 12 (80%)  23 (95.8%) | 3-20  1 (4.2%) | 0.1176 |
| **Hearing loss**  No  Yes | 23 (85.2%)  12 (100%) | 4 (14.8%)  0 (0%) | 0.1647 |
| **Performance status** | 82.5  [47.50-90]  (30-95) | 80  [65-95]  (5-100) | 0.9160 |
| **% of body surface covered with lesions** | 94  [62-100]  (40-95) | 94  [62-100]  (10-100) | 0.1316 |

IQR, Interquartile Range; n, Number of Participants; SOD (CISS), Social Diversion (Coping Inventory for Stressful Situations); VAS, Visual Analogue Scale.
